# Genome Sequence of WAU86/88-1, a New Variant of Vaccinia Virus Lister Strain from Poland

**DOI:** 10.1128/genomeA.01086-13

**Published:** 2014-01-09

**Authors:** Carla Mavian, Alberto López-Bueno, Antonio Alcamí

**Affiliations:** Centro de Biología Molecular Severo Ochoa (Consejo Superior de Investigaciones Científicas-Universidad Autónoma de Madrid), Campus de Cantoblanco, Madrid, Spain

## Abstract

The poxviruses Warsaw Agricultural University 86 (WAU86) and 88-1 (WAU88-1) were isolated in 1986 to 1988 from separate outbreaks in laboratory mice in Poland and described as ectromelia virus isolates. The genome sequences of these poxviruses reveal that they are almost identical and represent a novel variant of the vaccinia virus Lister strain.

## GENOME ANNOUNCEMENT

We report the genomic sequences of the poxvirus isolates Warsaw Agricultural University 86 (WAU86) and 88-1 (WAU88-1), isolated between 1986 and 1988 from laboratory mouse outbreaks in animal house facilities in Poland. These viruses were previously described as ectromelia virus (ECTV) isolates based on electron microscopy analysis, neutralization tests, and clinical symptoms in infected outbred white Swiss mice (1). WAU86 and WAU88-1 were obtained from M. Niemialtowski (Faculty of Veterinary Medicine, Warsaw Agricultural University, Warsaw, Poland). The viral particles were semipurified from infected monkey kidney epithelial cells (BSC-1) cells, and the viral genomes were agarose gel extracted before performing multiple displacement amplification with the Illustra GenomiPhi V2 (GE Healthcare) as described previously (2). Pyrosequencing on a GS-FLX (454 Life Sciences, Roche) of the viral genomes generated 28,072 reads for WAU88-1 and 117,669 reads for WAU86. The sequences were *de novo* assembled using Newbler 2.5.3 (454 Life Sciences, Roche) into two genomes with 51× and 242× coverages, respectively (3). These genomes are almost identical to each other, differing only in five deletions/insertions affecting homopolymers in the intergenic regions or pseudogenes that might correspond to typical errors of the 454 technology (4). We concluded that both genomes correspond to the same virus.

The genome of the virus, named WAU86/88-1, shows a typical orthopoxvirus A+T content (66.3%) and a length of 187,723 bp. Phylogenetic analysis based on the central conserved region of the genome (63 Kbp) revealed that this virus is related to vaccinia virus (VACV) strain Lister, commonly used in Europe during the smallpox eradication program (5). It has been reported that there is great genetic diversity of the vaccine strains used during the smallpox eradication campaign due to the production of vaccine stocks in the skin of animals that may not be natural hosts (5). For example, genome sequencing of two plaque-purified clones from VACV Dryvax differs in 573 polymorphisms and 53 deletions/insertions (6). Similarly, VACV-WAU86/88-1 differs from VACV Lister in 605 mismatches and several deletions/insertions, including one major deletion of 716 nucleotides in the inverted terminal repeats of the genome (7). Although WAU86 and WAU88-1 have been previously described as ECTV isolates, we found that the intranasal infection of five- to six-week-old female BALB/c mice with 10^7^ PFU of VACV-WAU86/88-1 did not cause mortalities or symptoms of disease, such us weight loss or skin lesions. This high attenuation of VACV-WAU86/88-1 was similar to that observed after infection of BALB/c mice with VACV Lister from Vestric Limited (A. Alcamí, unpublished data). The genetic classification of this virus as VACV Lister, together with its high attenuation in mice, questions the implications of WAU86 and WAU88-1 in the outbreaks in Poland. VACV Lister was used as a control in the characterization of these viruses, suggesting that the stocks of the poxvirus that caused the outbreaks may have been cross-contaminated with VACV Lister during their *in vitro* amplification (1). VACV Lister was originally supplied by Filczak from the Serum and Vaccines Research Laboratory (Warsaw, Poland), so it is conceivable that WAU86 and WAU88-1 correspond to the VACV Lister strain produced for the vaccination campaign in Poland (1).

### Nucleotide sequence accession number.

The genome sequence of VACV-WAU86/88-1 was submitted to GenBank with the accession no. KF866253.
